# Pharmacokinetics and efficacy of subcutaneous infliximab 120 mg every 2 weeks: a post hoc comparison with intravenous dosing in a phase 1 study in patients with inflammatory bowel disease

**DOI:** 10.1093/crocol/otag008

**Published:** 2026-01-27

**Authors:** Stefan Schreiber, Byong Duk Ye, Hyunseong Yu, Dong-Hyeon Kim, Young Nam Lee, Shomron Ben-Horin

**Affiliations:** Department for Internal Medicine I, University Hospital Schleswig-Holstein, Kiel University, Kiel, Germany; Department of Gastroenterology and Inflammatory Bowel Disease Center, University of Ulsan College of Medicine, Asan Medical Center, Seoul, Republic of Korea; Global Medical Division, Celltrion, Inc., Incheon, Republic of Korea; Global Medical Division, Celltrion, Inc., Incheon, Republic of Korea; Global Medical Division, Celltrion, Inc., Incheon, Republic of Korea; Gastroenterology Department, Sheba Medical Center and School of Medicine, Tel Aviv University, Tel-Hashomer, Israel

**Keywords:** infliximab, pharmacokinetics, standard dose, Crohn’s disease, ulcerative colitis

## Abstract

**Background:**

A phase 1 study of CT-P13 demonstrated pharmacokinetic non–inferiority of subcutaneous (SC) infliximab (IFX) to intravenous (IV) IFX in patients with inflammatory bowel disease. This post hoc analysis aimed to evaluate outcomes in the subset of patients who received IFX SC 120 mg every 2 weeks (Q2W) (eventual approved standard dose).

**Methods:**

In the phase 1 study (NCT02883452), patients received IFX IV induction therapy at Week (W) 0 and W2 and were randomized at W6 to receive IFX SC 120 mg (for patients weighing <80 kg at W6) or 240 mg (for ≥80 kg) Q2W from W6 through W54, or IFX IV 5 mg/kg every 8 weeks (Q8W) from W6 through W22 and IFX SC 120 mg (for <80 kg at W30) or 240 mg (for ≥80 kg) Q2W from W30 to W54. This analysis included patients weighing <80 kg and thus received IFX SC 120 mg Q2W from W6 (SC 120 mg Q2W subset, *n* = 48) or W30 (IV 5 mg/kg Q8W subset, *n* = 45). Intense pharmacokinetic monitoring was used to compare the 2 subsets between W22 and W30.

**Results:**

Between W23 and W25, serum IFX concentrations in both subsets were within a similar range, but from W25 through W30, patients in the SC 120 mg Q2W subset exhibited higher serum IFX concentrations than in the IV 5 mg/kg Q8W subset, yielding significantly higher drug exposure. Efficacy and safety outcomes were comparable between subsets throughout.

**Conclusions:**

These data support a favorable pharmacokinetic profile of the approved standard dose of IFX SC.

## Graphical abstract

**Graphical Abstract:**
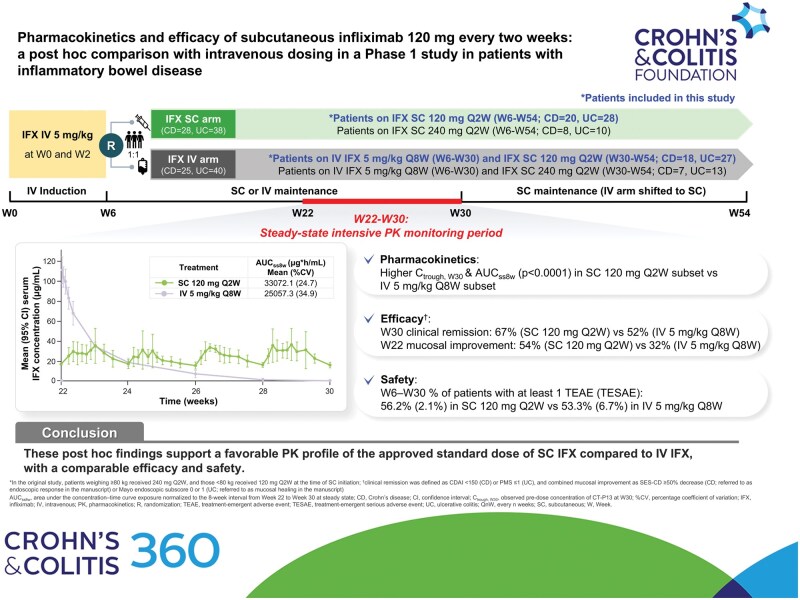

## Introduction

Infliximab (IFX) was the first anti–tumor necrosis factor drug approved for the treatment of inflammatory bowel disease (IBD) in 1998 and, since then, has helped revolutionize clinical outcomes for patients with Crohn’s disease (CD) and ulcerative colitis (UC).^1^^,^^2^

For more than 20 years, IFX was administered solely via intravenous (IV) infusion, ignoring the half-life time of the drug which is between 11 and 19 days.^3^ In 2020, a subcutaneous (SC) formulation of IFX was approved in Europe for patients with IBD^4^^,^^5^ and is administered in intervals (every 2 weeks [Q2W]) that approximate the half-life time of the therapeutic antibody. IFX SC was initially approved by the European Medicines Agency (EMA)^5^ for the rheumatoid arthritis indication, with its use later expanded to UC and CD based on the results of the phase 1 1.6 study.^6^ The US Food and Drug Administration (FDA)^7^ subsequently also approved IFX SC via the new drug approval pathway, supported by clinical trial evidence from two phase 3, placebo-controlled maintenance studies, LIBERTY-CD and LIBERTY-UC.^8^ SC administration of IFX Q2W results in target coverage with less variations, and in higher trough drug levels, than IV administration every 8 weeks (Q8W), without changing the known safety profile.^6^^,^^9^ In addition, IFX SC expands treatment options for patients, with the added benefits of self-administration and greater convenience by reducing medical visits.^10^

The 1.6 study remains the only randomized controlled trial (RCT) to directly compare SC and IV administration of IFX in a population of patients with IBD.^6^ The dose of IFX SC administered in part 2 of the 1.6 study was guided by a dose-finding part 1 of the study and finally determined by body weight, with those weighing <80 kg given 120 mg Q2W and those ≥80 kg given 240 mg Q2W.^6^^,^^11^^,^^12^ Final approval by the EMA was granted for the 120 mg SC Q2W dosage in patients with IBD,^5^^,^^7^ supported by population pharmacokinetic (PK) modelling, which showed that patients with IBD receiving this dosage achieved higher pre-dose serum IFX levels and non–inferior efficacy compared with the IV formulation, including those with higher body weight (up to 150 kg).^4^ However, previously reported data from the 1.6 study^6^ presented a pooled analysis of patients receiving either the standard SC 120 mg dose or the SC 240 mg dose, and thus may not reflect the current FDA label, which supports the regimen of 120 mg Q2W regardless of body weight. Additionally, the PK profile of IFX SC, which has been simulated in comparison with IFX IV based on modelling data,^13^ has not been examined using real-world data. There is, therefore, a growing demand for comparative detailed PK data between SC and IV standard dosing in the clinical field.

This brief communication aims to provide data focusing on the subset of patients with IBD who received the now approved dosing of IFX SC 120 mg Q2W in part 2 of the 1.6 study.

## Materials and methods

### Study design

This was a post hoc analysis of the 1.6 study, the design of which has been previously published.^6^ Briefly, this was an open-label, randomized, parallel-subset, phase 1 study to evaluate PK, efficacy, and safety of IFX SC and IFX IV in patients with moderate to severe CD or UC (key eligibility criteria are described in Supplementary Methods). During the induction phase (Week [W] 0-6), all patients received IFX IV 5 mg/kg at W0 and W2. At W6, patients who had received 2 full doses of IFX IV without safety concerns (per investigator’s judgment) were randomized (1:1) to either the IFX SC (*n* = 66) or IFX IV arm (*n* = 65). During the maintenance phase (W6-W54), patients in the IFX SC arm received, depending on their body weight, either IFX SC 120 mg Q2W (<80 kg) or 240 mg Q2W (≥80 kg). In the IFX IV arm, patients received IFX IV 5 mg/kg Q8W from W6 through W22. Clinical observation continued until W30, that is, for 8 weeks after the last IFX IV dose. At W30, patients in the IFX IV arm shifted to IFX SC 120 mg Q2W or 240 mg Q2W, depending on their body weight. This post hoc analysis included patients weighing <80 kg and thus received IFX SC 120 mg Q2W from W6 (SC 120 mg Q2W subset, *n* = 48) or W30 (IV 5 mg/kg Q8W subset, *n* = 45). The IV 5 mg/kg Q8W subset comprises patients who received IFX IV 5 mg/kg Q8W and then shifted to IFX SC 120 mg Q2W at W30 until the end of the study.

### Study endpoints

For the current analysis, PK, efficacy, and safety were compared between the SC 120 mg Q2W and IV 5 mg/kg Q8W subsets before and after patients in the IV 5 mg/kg Q8W subset shifted to IFX SC 120 mg Q2W at W30. PK endpoints included serum IFX concentration; model-predicted area under the concentration–time curve at steady state (AUC_τ_); model-predicted area under the concentration–time curve exposure normalized to the 8-week ­interval from W22 to W30 at steady state (AUC_ss8w_); model-predicted trough IFX serum concentration (C_trough_); and maximum IFX serum concentration at steady state (C_max, ss_). Efficacy endpoints were defined as follows: clinical response (CD: ≥100-point decrease from baseline in Clinical Disease Activity Index [CDAI] score; UC: ≥2-point decrease from baseline in partial Mayo score [PMS] with accompanying ≥1-point decrease in rectal bleeding subscore, or an absolute rectal bleeding subscore of 0 or 1), clinical remission (CD: absolute CDAI score of <150 points; UC: PMS of ≤1 point), endoscopic response (CD only: ≥50% decrease from baseline in Simplified Endoscopic Activity Score for CD [SES-CD]), and mucosal healing (UC only: absolute endoscopic subscore of 0 or 1 per the Mayo scoring system). Concentrations of fecal calprotectin (FC) and C-reactive protein (CRP), measured throughout the study, were presented for a pooled population of patients with CD and UC. Safety data were assessed for selected adverse events. Adverse events of special interest included administration-related reactions (classified as infusion-related reactions [IRRs; following IFX IV administration], systemic injection reactions [SIRs; following IFX SC administration], or delayed hypersensitivity reactions), ­localized injection-site reactions (ISRs), infection, and ­malignancy. Study assessments are described in Supplementary Methods.

### Statistical analysis

Continuous variables were summarized as means with 95% confidence intervals (CIs) unless otherwise noted. The proportions of patients meeting the efficacy endpoint criteria (clinical response and remission, endoscopic response, and mucosal healing) were calculated and compared between the 2 patient subsets.

Statistical analyses of PK parameters and clinical outcomes up to W54 were conducted using R v4.4.1 (R Foundation for Statistical Computing). Descriptive statistical significance was assessed using Fisher exact test for dichotomous variables or Wilcoxon rank-sum test for continuous variables. Analyses were descriptive, and nominal *P*-values are reported.

A population PK model was used to estimate PK parameters (except C_trough, W22_) for individual patients using a non–linear mixed-effects PK model. PK parameters were obtained using both NONMEM v7.3.0 (ICON Development Solutions) and R v3.4.1.

### Ethical considerations

Ethics approval for the CT-P13 1.6 study has been published previously.^6^ All patients provided written informed consent. The trial is registered at ClinicalTrials.gov: NCT02883452.

## Results

### Patients

Forty-eight patients (20 with CD, 28 with UC) were treated with the approved standard dose of IFX SC 120 mg Q2W from W6 through W54 (SC 120 mg Q2W subset), while 45 patients (18 with CD, 27 with UC) were treated with IFX IV 5 mg/kg Q8W (W6-W22) then IFX SC 120 mg Q2W (W30-W54) (IV 5 mg/kg Q8W subset). The median (range) body weight of patients in the 2 subsets were 62.7 (45.2-79.0) kg and 62.9 (43.2-78.0) kg, respectively. Baseline characteristics were generally comparable between subsets (Table 1).

**Table 1:** Patient demographics and disease characteristics.

Parameter	SC 120 mg Q2W subset (*n* = 48)	**IV 5 mg/kg Q8W subset** ^a^ **(*n* = 45)**
**Patient demographics**		
**Age (years), median (range)**	32 (18-69)	35 (18-70)
**Sex, *n* (%)**		
** Male**	23 (47.9)	18 (40.0)
** Female**	25 (52.1)	27 (60.0)
**Race, *n* (%)**		
** Asian**	2 (4.2)	4 (8.9)
** White**	45 (93.8)	41 (91.1)
** Other**	1 (2.1)	0
**Ethnicity, *n* (%)**		
** Hispanic or Latino**	2 (4.2)	0
** Non–Hispanic/non–Latino**	46 (95.8)	45 (100)
** Unknown**	0	0
**Height (cm), median (range)**	168 (144-186)	169 (157-193)
**Weight (kg), median (range)**	62.7 (45.2-79.0)	62.9 (43.2-78.0)
**BMI (kg/m^2^), median (range)**	22.5 (16.4-28.2)	22.0 (15.3-27.9)
**Disease characteristics**		
**Disease, *n* (%)**		
** CD**	20 (41.7)	18 (40.0)
** UC**	28 (58.3)	27 (60.0)
**CDAI score (patients with CD), mean (SD)**	286.8 (49.3)	294.0 (62.9)
**SES-CD (patients with CD), mean (SD)**	9.6 (7.8)	8.3 (6.1)
**TMS (patients with UC), mean (SD)**	7.8 (1.7)	7.9 (1.7)
**PMS (patients with UC), mean (SD)**	5.3 (1.4)	5.8 (1.3)
**Current treatment with AZA, 6-MP, or MTX, *n* (%)**		
** Yes**	20 (41.7)	22 (48.9)
** No**	28 (58.3)	23 (51.1)
**Clinical response^b^ at Week 6, *n* (%)**		
** Yes**	36 (75.0)	35 (77.8)
** No**	12 (25.0)	10 (22.2)

a Patients in the IV 5 mg/kg Q8W subset shifted to IFX SC 120 mg Q2W at Week 30.

b Assessed using PMS (≥2-point decrease in PMS with accompanying ≥1-point decrease in rectal bleeding subscore, or an absolute rectal bleeding subscore of 0 or 1) for patients with UC, and a 70-point decrease in CDAI score for patients with CD.

Abbreviations: 6-MP, 6-mercaptopurine; AZA, azathioprine; BMI, body mass index; CD, Crohn’s disease; CDAI, Clinical Disease Activity Index; IFX, infliximab; IV, intravenous; MTX, methotrexate; PMS, partial Mayo score; QnW, every *n* weeks; SC, subcutaneous; SD, standard deviation; SES-CD, Simplified Endoscopic Activity Score for Crohn’s Disease; TMS, total Mayo score; UC, ulcerative colitis.

### Pharmacokinetics

**Figure 1:**
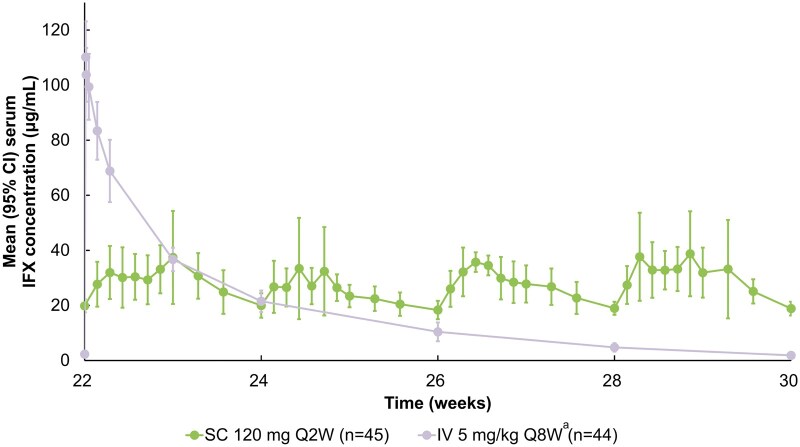
Mean (95% CI) serum IFX concentrations in the two subsets during the intensive PK monitoring period. ^a^Patients in the IV 5 mg/kg Q8W subset shifted to IFX SC 120 mg Q2W at Week 30. PK were analyzed in patients who received at least one full dose of study drug at Week 6 or thereafter and had at least one post-Week 6 PK concentration result. CI, confidence interval; IFX, infliximab; IV, intravenous; PK, pharmacokinetic; QnW, every *n* weeks; SC, subcutaneous.

Serum IFX concentrations during the intensive PK monitoring period (W22-W30), corresponding to 1 treatment cycle in the IV arm (8 weeks), are presented in Figure 1. By W23-W25, serum IFX concentrations in both subsets were within a similar range, but from W25 through W30, the SC 120 mg Q2W subset exhibited higher serum IFX concentrations than the IV 5 mg/kg Q8W subset, which was supported by non–overlapping 95% CIs. The SC 120 mg Q2W subset maintained stable serum IFX levels >18 μg/mL throughout this period.

Model-predicted PK outcomes during the intensive PK monitoring period are shown in Table S1. The model-predicted mean C_max, ss_ after dose administration was significantly higher in the IV 5 mg/kg Q8W subset than in the SC 120 mg Q2W subset at W22 (*P* < .0001). The model-predicted mean AUC_ss8w_ was statistically significantly higher in the SC 120 mg Q2W subset (33,072.1 μg*h/mL) than in the IV 5 mg/kg Q8W subset (25,057.3 μg*h/mL) (*P* < .0001).

Mean pre–dose serum IFX concentrations over the full course of the study are shown in Figure S1. From W14 to W30 of the maintenance phase, the pre–dose serum IFX concentrations were statistically significantly higher in the SC 120 mg Q2W subset than in the IV 5 mg/kg Q8W subset (*P* < .0001). Following the IV-to-SC shift at W30, a statistically significant difference in mean pre–dose serum IFX concentrations was observed between subsets up to W38; however, this significance disappeared after W46 (*P* = .051).

### Efficacy

Selected efficacy outcomes are shown in Figure S2. In patients with CD, rates of clinical response and clinical remission, as assessed by CDAI, were generally comparable between the SC 120 mg Q2W and IV 5 mg/kg Q8W subsets (Figure S2A). While the rates at W46 showed some numerical differences, the differences were not statistically significant (*P* = .096 and *P* = .1014 for clinical response and clinical remission, respectively). By W54, the proportions were similar in the 2 subsets. In patients with UC, rates of clinical response and clinical remission, as assessed by PMS, were generally comparable between subsets (Figure S2B). In patients with CD, the rate of endoscopic response at W22 was 81.8% in the SC 120 mg Q2W subset and 40.0% in the IV 5 mg/kg Q8W (Figure S2C). By W54, following the IV-to-SC shift at W30, rates in the IV 5 mg/kg Q8W subset had increased, resulting in similar outcomes between subsets.

In patients with UC, mucosal healing rates were 42.9% in the SC 120 mg Q2W subset and 30.8% in the IV 5 mg/kg Q8W subset at W22 (Figure S2D). Following the IV-to-SC shift at W30, rates in the 2 subsets had increased to similar levels by W54.

The mean actual values for efficacy parameters are shown in Figure S3. In patients with CD, mean CDAI scores were comparable in the 2 subsets throughout the analysis period (Figure S3A). In patients with UC, mean PMS were slightly lower in the SC 120 mg Q2W than in the IV 5 mg/kg Q8W subset during W14-W30 (Figure S3B). After the IV-to-SC shift at W30, the scores in the 2 subsets became similar.

Mean (95% CI) concentrations of FC and CRP throughout the study are shown in Figure S4. Excluding a single outlier CRP result of 8.40 mg/dL in the IV arm at W30, FC and CRP results were largely comparable between subsets throughout the study.

### Safety

Safety data for the full study population have been previously published.^6^ In the current subset analysis, the safety findings for the SC 120 mg Q2W subset remain comparable with those for the IV 5 mg/kg Q8W subset, and no new safety signals were identified during W6 to <W30 or from W30 onwards (Tables S2 and S3 and Supplementary Results). The incidence of localized ISRs was 4.4% from W30 onwards in patients who shifted from IV to SC IFX. In the SC 120 mg Q2W subset, the incidence of ISRs was 16.7% during W6 to <W30 and 13.3% from W30 onwards, showing a relatively steady rate throughout the maintenance phase.

## Discussion

This brief report details a post hoc comparison of the SC 120 mg Q2W and IV 5 mg/kg Q8W subsets in part 2 of the 1.6 study^6^ with regards to PK, efficacy, and safety in IBD. In contrast to the original publication, which reported aggregated data from the 2 doses administered (120 and 240 mg), the present analysis focused only on patients with body weight <80 kg who received the eventual approved standard dose of IFX SC (120 mg Q2W) and assessed serum IFX levels during the intensive PK monitoring period from W22 to W30. Our results support predictions from a previous simulation of the PK of IFX SC versus IFX IV.^13^ Additionally, in the current subset analysis, higher pre-dose serum IFX levels were observed in patients in the SC 120 mg Q2W than in the IV 5 mg/kg Q8W subset prior to the IV-to-SC shift (W0-W30) as well as a higher drug-exposure (AUC) during 8 weeks of maintenance. Recent real-world studies have also indicated higher C_trough_ with SC versus IV administration, suggesting potential benefits in terms of long-term efficacy and possibly less need for combination therapy with immunomodulators,^14^ although it should not be assumed that the clinical implications of C_trough_ levels are identical with SC versus IV formulations.^15^^,^^16^ This is consistent with the results of this subset analysis. Collectively, these findings are significant in reaffirming a better PK profile with IFX SC compared with the IV formulation, even when selectively examining the approved lower dose. The study also validated the accuracy of modelling data and may help to predict the PK behavior and efficacy outcomes in patients receiving standard-dose IFX SC.

A key strength of this study is that the data analyzed were obtained through an RCT that included efficacy outcomes comparing IFX administration routes. It is the only prospective study using steady-state PK sampling in patients with IBD. Limitations of the study include that it was not designed, and therefore not statistically powered, to specifically investigate patients receiving the standard dose of IFX SC as a subgroup. A lack of statistical power, due to the post hoc nature of this analysis, might explain the absence of statistical difference observed between groups in efficacy outcomes, in contrast to other real-world data indicating better long-term outcomes with SC relative to IV IFX.^14^ Therefore, the analysis should be considered descriptive. Moreover, patients receiving standard-dose IFX SC weighed <80 kg as per the design of the primary RCT, which may limit the generalizability of the results to a real-world setting.

## Conclusions

In conclusion, this post hoc analysis of rigorously sampled patients with body weight <80 kg receiving the approved standard dose of IFX SC (120 mg Q2W) provides evidence of higher IFX drug exposure and C_trough_ levels in patients with IBD compared with the IV formulation. The study demonstrates superior coverage of the target by adjusting IFX dosing to the half-life time. The findings reported herein for the final approved standard dose confirm the assumptions from the phase 1 study. While the data are comparable to real-world treatment, further prospective studies are needed to determine the rules for individual dosing adaptations.^14^

## Supplementary Material

otag008_Supplementary_Data

## Data Availability

A redacted study protocol has been published as part of the ­Supplementary Materials for an earlier publication.^6^ Individual ­participant data will not be shared.
