# Supplementary material for: Pharmacokinetics and efficacy of subcutaneous infliximab 120 mg every 2 weeks: a post hoc comparison with intravenous dosing in a phase 1 study in patients with inflammatory bowel disease
Source: Crohns Colitis 360. 2026 Jan 27;8(1):otag008. doi: 10.1093/crocol/otag008 (PMC13006203; doi:10.1093/crocol/otag008)
Supplement: otag008_Supplementary_Data [file otag008_supplementary_data.docx]

# Supplementary material

Pharmacokinetics and efficacy of subcutaneous infliximab 120 mg every 2 weeks: a post hoc comparison with intravenous dosing in a Phase 1 study in patients with inflammatory bowel disease

## Supplementary Methods

## *Patients*

Full eligibility criteria have been published [[1](#_ENREF_1)]. Briefly, patients were aged 18–75 years, with active ulcerative colitis (UC) or Crohn’s disease (CD), had disease duration of ≥3 months before first administration of study drug (Day 0), were tumor necrosis factor inhibitor naïve, and had received no prior biologics for UC or CD. Patients with tuberculosis exposure or diagnosis were excluded. Patients had received conventional therapy for active UC (corticosteroids alone or in combination with thiopurines and 5-aminosalicylates) or CD (corticosteroids and/or immunomodulators) but had not responded despite an adequate course of therapy. Immunomodulators were allowed if stable doses were received for ≥8 weeks (thiopurines) or ≥6 weeks (methotrexate) before first administration of study drug (Day 0) and were maintained throughout the study.

## *Study assessments*

Blood samples for pharmacokinetic (PK) assessment were obtained at all study visits from Week 0 through Week 54, including during the intensive PK monitoring period occurring between Weeks 22 and 30. In addition, during intensive PK monitoring, patients in the SC 120 mg every 2 weeks (Q2W) subset were randomized (1:1:1:1) to groups A, B, C, or D for frequent blood sampling (every 24 hours for the first 7 days, then at Day 9 and Day 11) following the scheduled dose at Weeks 22, 24, 26, or 28, respectively. Serum infliximab (IFX) levels were measured quantitatively using electrochemiluminescence (Meso Scale Discovery, Meso Scale Diagnostics LLC, Rockville, MD). All endoscopy data were assessed by central review, conducted by independent reviewers blinded to treatment allocations, using a paired read with adjudication algorithm.

## Supplementary Results

***Safety***

**Week 6 to <30**: Treatment-emergent adverse events (TEAEs) were reported by 27 (56.2%) patients in the SC 120 mg Q2W subset and 24 (53.3%) patients in the IV 5 mg/kg Q8W subset (P=0.8364), with study drug–related TEAEs reported by 16 (33.3%) and 9 (20.0%) patients, respectively (P=0.1671). Treatment-emergent serious adverse events (TESAEs) were reported by 1 (2.1%) patient in the SC 120 mg Q2W subset and 3 (6.7%) patients in the IV 5 mg/kg Q8W subset (P=0.3510). TEAEs leading to study drug discontinuation were reported by 1 (2.1%) patient in the IV 5 mg/kg Q8W subset only. No deaths were reported during the study.

**Week ≥30**: TEAEs were reported by 24 (50.0%) patients in the SC 120 mg Q2W subset and 15 (33.3%) patients in the IV 5 mg/kg Q8W subset, who shifted to IFX SC 120 mg Q2W at Week 30 (P=0.1412); correspondingly, 10 (20.8%) and 8 (17.8%) patients reported study drug–related TEAEs. Similar proportions of patients reported TESAEs in each subset (SC 120 mg Q2W: 3 [6.3%] patients; IV 5 mg/kg Q8W: 3 [6.7%] patients). One (2.1%) patient in the SC 120 mg Q2W subset experienced a TEAE leading to study drug discontinuation.

## Supplementary Tables

## Supplementary Table 1 Model-predicted pharmacokinetic parameters

| **Parameter, mean (%CV)** | **SC 120 mg**  **Q2W subset**  **(n=45)** | **IV 5 mg/kg Q8W subset^a^**  **(n=44)** | **Nominal  P value** |
| --- | --- | --- | --- |
| **AUC_τ_ (μg*h/mL)** |  |  |  |
| Week 22 | 7935.0 (36.5) | 24850.5 (34.4) | <0.0001 |
| Week 24 | 7370.0 (23.2) | N/A |  |
| Week 26 | 8939.1 (9.7) | N/A |  |
| Week 28 | 8631.8 (25.2) | N/A |  |
| **AUC_ss8w_ (μg*h/mL)** |  |  |  |
| Week 22 | 33072.1 (24.7) | 25057.3 (34.9) | <0.0001 |
| **C_trough_ (μg/mL)** |  |  |  |
| Week 22 | 18.9 (38.9) | 2.2 (80.4) | <0.0001 |
| Week 24 | 17.9 (30.9) | N/A |  |
| Week 26 | 19.3 (23.8) | N/A |  |
| Week 28 | 18.8 (36.0) | N/A |  |
| **C_max,ss_ (μg/mL)** |  |  |  |
| Week 22 | 28.9 (31.7) | 97.6 (20.7) | <0.0001 |
| Week 24 | 25.1 (24.3) | N/A |  |
| Week 26 | 30.1 (7.5) | N/A |  |
| Week 28 | 28.6 (22.3) | N/A |  |

During intensive PK monitoring, patients in the SC 120 mg Q2W subset were randomized (1:1:1:1) to groups A, B, C, or D for frequent blood sampling (every 24 hours for the first 7 days, then at Day 9 and Day 11) following the scheduled dose at Weeks 22, 24, 26, or 28, respectively. For SC 120 mg Q2W, model-predicted AUCτ and model-predicted C_max,ss_ were evaluated in one of the four subset groups at each time point; model-predicted and observed C_trough_ were evaluated in two of the four subset groups at Weeks 22, 24, and 26.

^a^Patients in the IV 5 mg/kg Q8W subset shifted to IFX SC 120 mg Q2W at Week 30.

PK were analyzed in patients who received at least one full dose of study drug at Week 6 or thereafter and had at least one post-Week 6 PK concentration result.

%CV, percentage coefficient of variation; AUCτ, area under the concentration–time curve at steady state; AUC_ss8w_, area under the concentration–time curve exposure normalized to the 8-week interval from Week 22 to Week 30 at steady state; C_max,ss_, maximum IFX serum concentration at steady state; C_trough_, trough IFX serum concentration; IFX, infliximab; IV, intravenous; N/A, not applicable; PK, pharmacokinetic; QnW, every n weeks; SC, subcutaneous.

## Supplementary Table 2 Summary of safety outcomes by number of adverse events during the maintenance phase

|  | **Week 6 to <30** | | **Week ≥30** | |
| --- | --- | --- | --- | --- |
| **Total number of adverse events** | **SC**  **120 mg Q2W subset (n=48)** | **IV  5 mg/kg Q8W subset (n=45)** | **SC**  **120 mg Q2W subset (n=48)** | **IV  5 mg/kg Q8W subset ^a^ (n=45)** |
| **TEAEs** | 71 | 55 | 84 | 48 |
| **TESAEs** | 1 | 3 | 3 | 5 |
| **TEAEs leading to study drug discontinuation** | 0 | 1 | 1 | 0 |
| **TEAEs classified as IRR** | N/A | 2 | N/A | N/A |
| **TEAEs classified as SIR** | 0 | N/A | 1 | 0 |
| **TEAEs classified as delayed hypersensitivity** | 1 | 0 | 0 | 0 |
| **TEAEs classified as localized ISR** | 16 | 0 | 16 | 13 |
| **TEAEs classified as infection** | 11 | 8 | 16 | 10 |
| **TEAEs classified as malignancy** | 0 | 0 | 1 | 0 |

^a^Patients in the IV 5 mg/kg Q8W subset shifted to IFX SC 120 mg Q2W at Week 30.

IFX, infliximab; IRR, infusion-related reaction; ISR, injection-site reaction; IV, intravenous;
N/A, not available; QnW, every n weeks; SC, subcutaneous; SIR, systemic injection reaction;
TEAE, treatment-emergent adverse event.

## Supplementary Table 3 Summary of safety outcomes by number of patients who experienced ≥1 adverse event during the maintenance phase

|  | **Week 6 to <30** | | **Week ≥30** | |
| --- | --- | --- | --- | --- |
| **n (%)** | **SC**  **120 mg Q2W subset (n=48)** | **IV  5 mg/kg Q8W subset (n=45)** | **SC**  **120 mg Q2W subset (n=48)** | **IV  5 mg/kg Q8W subset ^a^ (n=45)** |
| **Patients with ≥1 TEAE** | 27 (56.2) | 24 (53.3) | 24 (50.0) | 15 (33.3) |
| Study drug–related | 16 (33.3) | 9 (20.0) | 10 (20.8) | 8 (17.8) |
| Study drug–unrelated | 19 (39.6) | 21 (46.7) | 20 (41.7) | 11 (24.4) |
| **Patients with ≥1 TESAE** | 1 (2.1) | 3 (6.7) | 3 (6.3) | 3 (6.7) |
| Study drug–related | 0 | 0 | 1 (2.1) | 2 (4.4) |
| Study drug–unrelated | 1 (2.1) | 3 (6.7) | 2 (4.2) | 2 (4.4) |
| **Patients with ≥1 TEAE leading to study drug discontinuation** | 0 | 1 (2.2) | 1 (2.1) | 0 |
| Study drug–related | 0 | 1 (2.2) | 1 (2.1) | 0 |
| **Patients with ≥1 TEAE classified as IRR** | N/A | 2 (4.4) | N/A | N/A |
| **Patients with ≥1 TEAE classified as SIR** | 0 | N/A | 1 (2.1) | 0 |
| **Patients with ≥1 TEAE classified as delayed hypersensitivity** | 1 (2.1) | 0 | 0 | 0 |
| **Patients with ≥1 TEAE classified as localized ISR** | 8 (16.7) | 0 | 6 (13.3) | 2 (4.4) |
| **Patients with ≥1 TEAE classified as infection** | 8 (16.7) | 7 (15.6) | 9 (18.8) | 7 (15.6) |
| **Patients with ≥1 TEAE classified as malignancy** | 0 | 0 | 1 (2.1) | 0 |

^a^Patients in the IV 5 mg/kg Q8W subset shifted to IFX SC 120 mg Q2W at Week 30.

IFX, infliximab; IRR, infusion-related reaction; ISR, injection-site reaction; IV, intravenous;
N/A, not available; QnW, every n weeks; SC, subcutaneous; SIR, systemic injection reaction; TEAE, treatment-emergent adverse event; TESAE, treatment-emergent serious adverse event.

## Supplementary Fig. 1 Mean (95% CI) pre-dose serum IFX concentrations in the two patient subsets


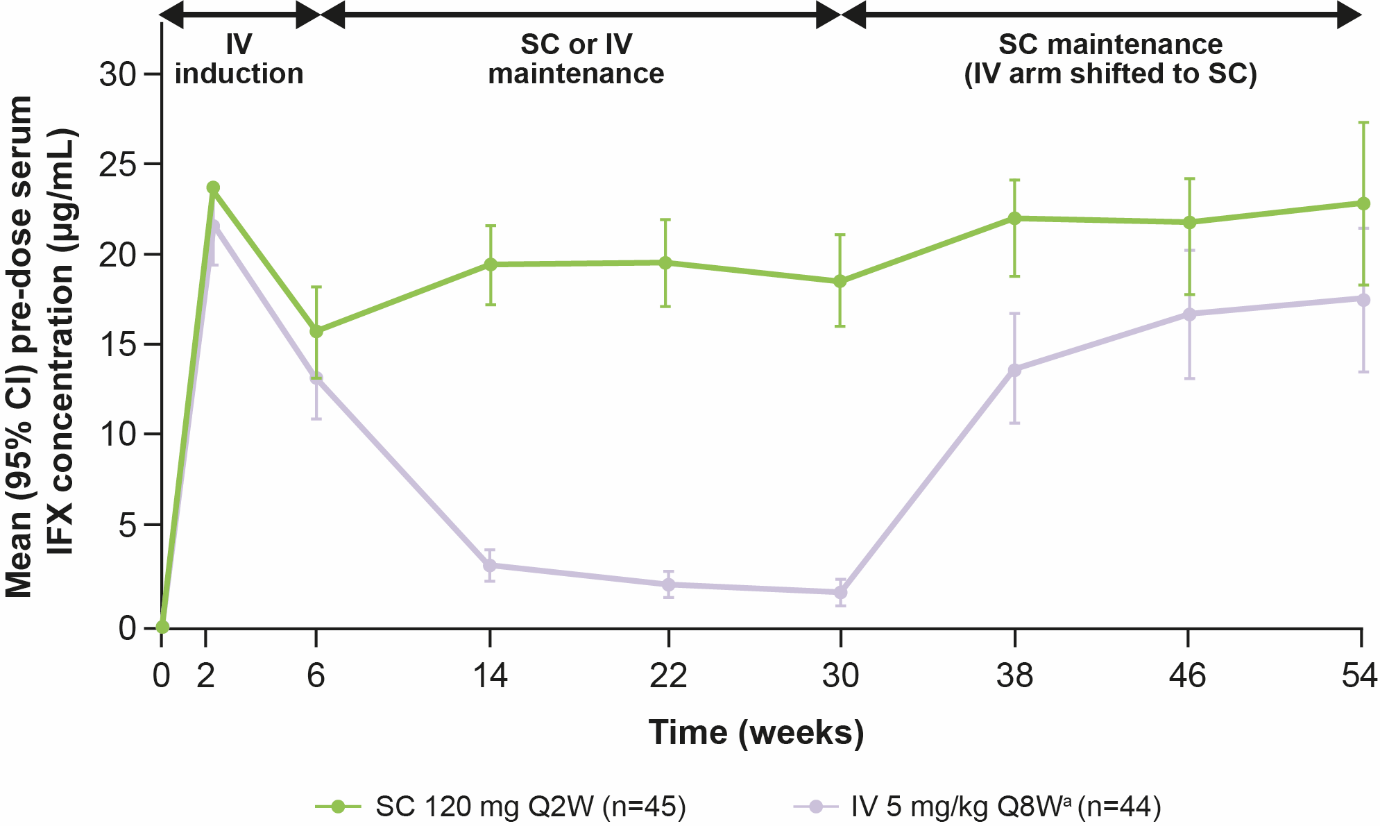


^a^Patients in the IV 5 mg/kg Q8W subset shifted to IFX SC 120 mg Q2W at Week 30.

PK were analyzed in patients who received at least one full dose of study drug at Week 6 or thereafter and had at least one post-Week 6 PK concentration result.

CI, confidence interval; IFX, infliximab; IV, intravenous, QnW, every n weeks; SC, subcutaneous.

## Supplementary Fig. 2 Clinical response, clinical remission in CD and UC, endoscopic response in CD, and mucosal healing in UC. Proportion of patients achieving clinical response and clinical remission by (a) CDAI score in CD and (b) PMS in UC. (c) Proportion of patients with CD achieving endoscopic response by SES-CD (d). Proportion of patients with UC achieving mucosal healing by endoscopic subscore per the Mayo scoring system


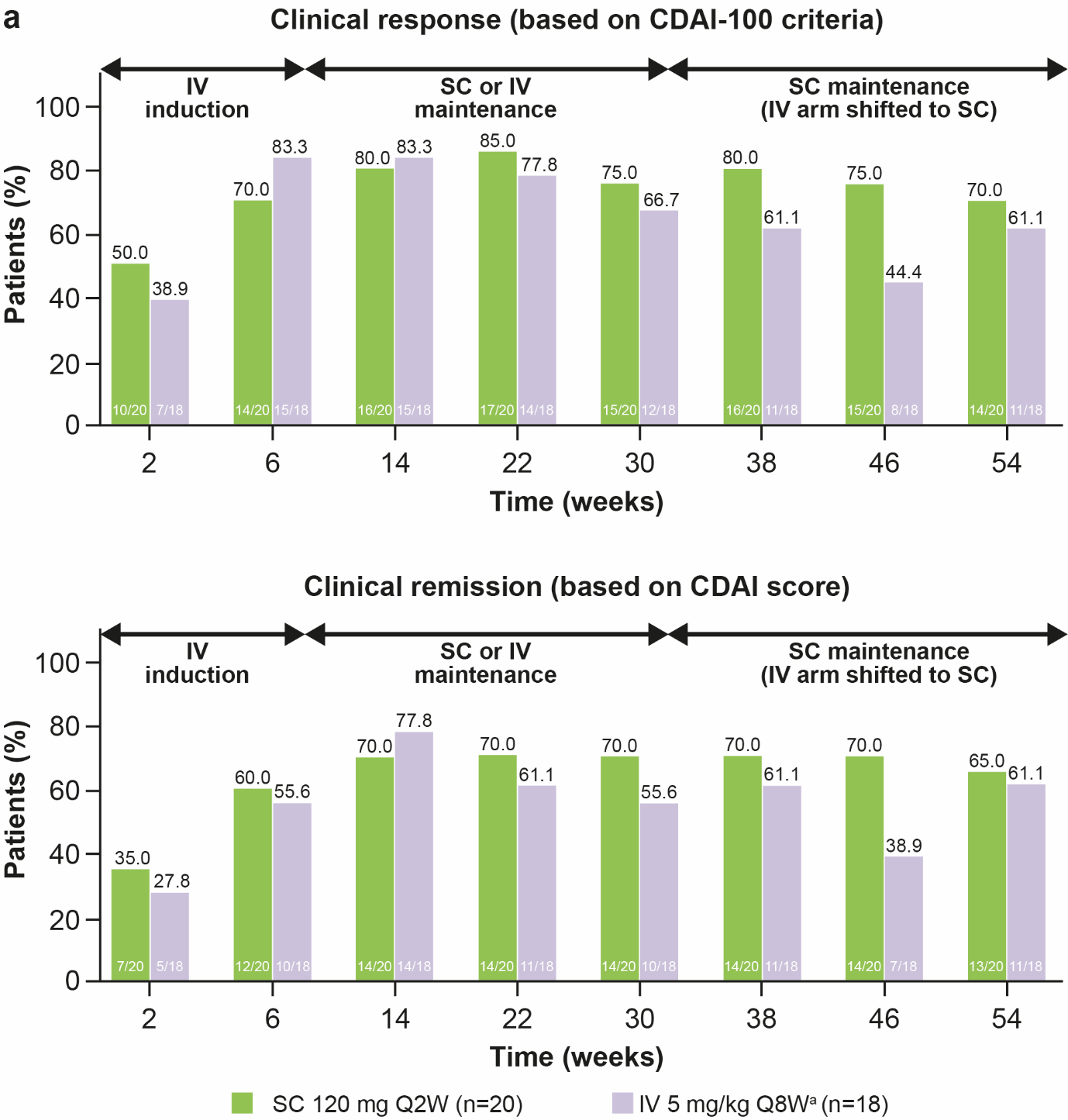


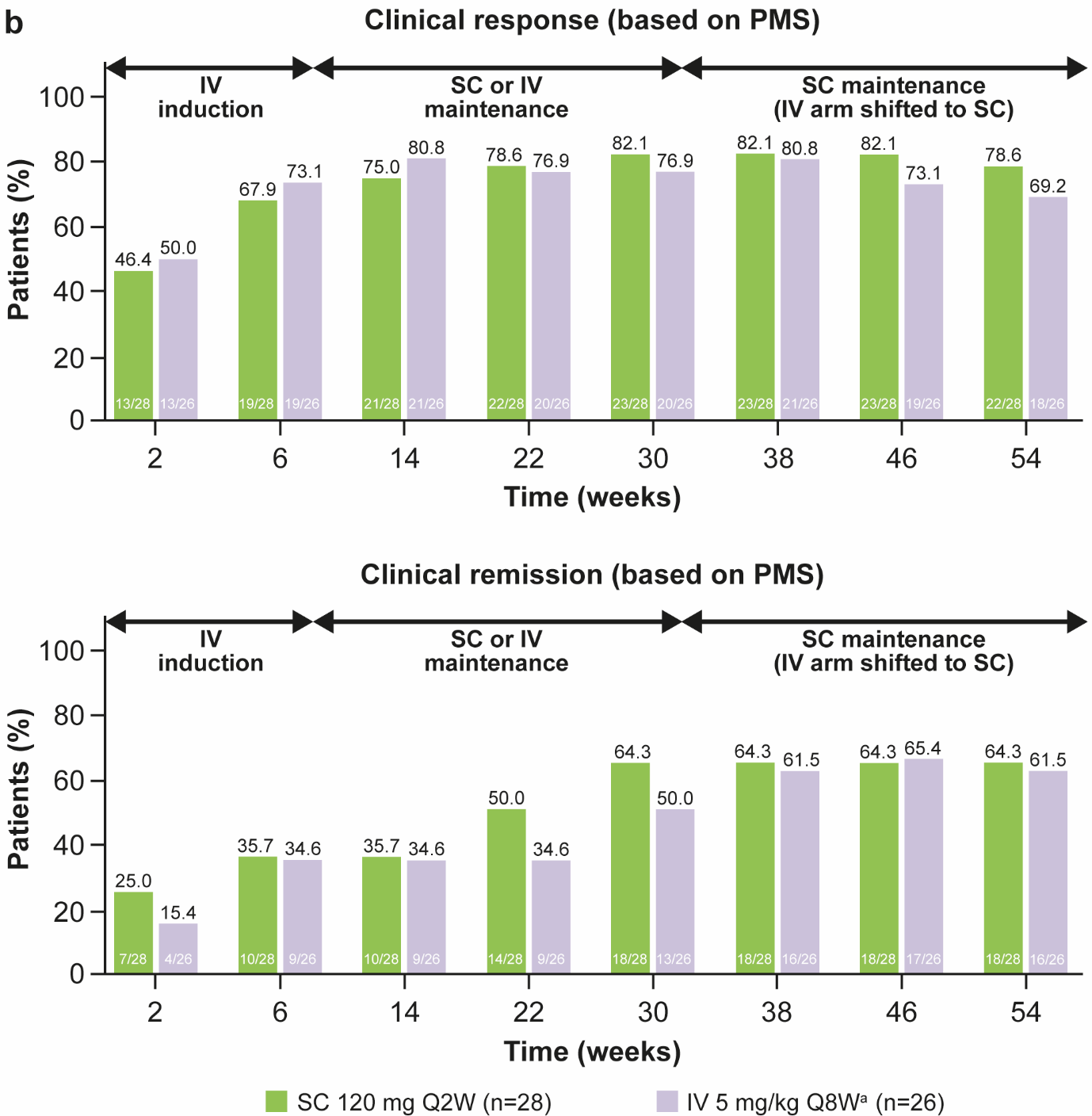


**
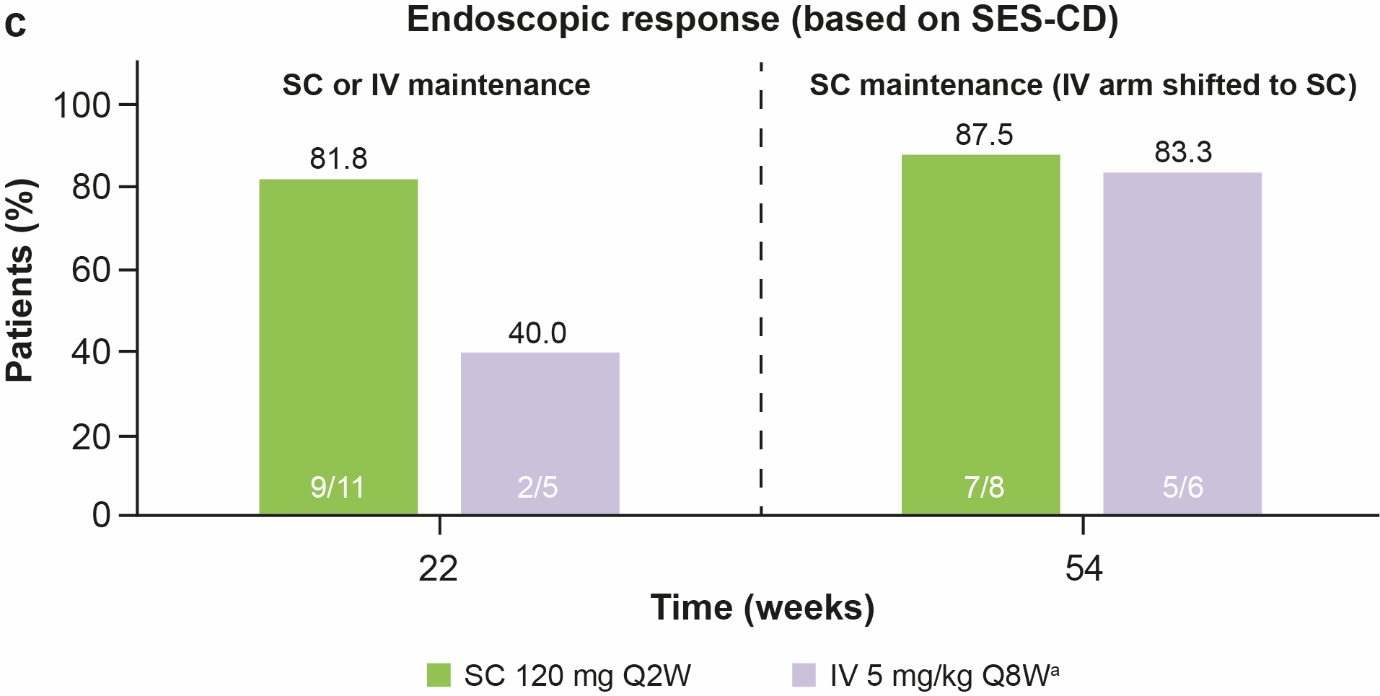
**

**
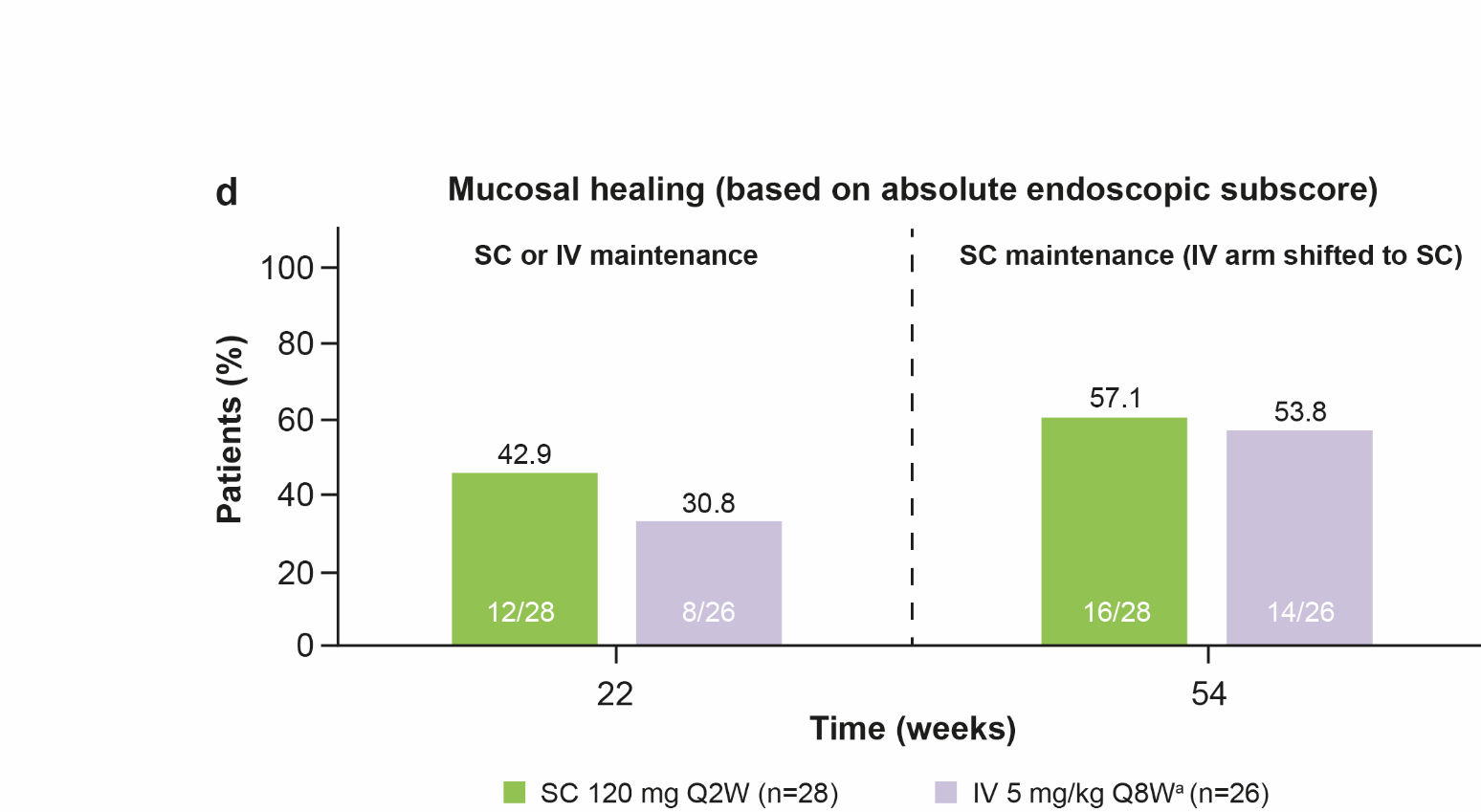
**

CD: CDAI-100 response (≥100-point decrease from baseline in CDAI score); clinical remission (absolute CDAI score of <150 points); endoscopic response (≥50% decrease from baseline in SES-CD).
UC: clinical response (≥2-point decrease from baseline in PMS with accompanying ≥1-point decrease in rectal bleeding subscore, or an absolute rectal bleeding subscore of 0 or 1); clinical remission (PMS of ≤1 point); mucosal healing (absolute endoscopic subscore of 0 or 1 per the Mayo scoring system).

^a^Patients in the IV 5 mg/kg Q8W subset shifted to IFX SC 120 mg Q2W at Week 30.

Efficacy was analyzed in patients who received at least one full dose of study drug at Week 6 or thereafter and had at least one post-Week 6 efficacy assessment result.

CD, Crohn’s disease; CDAI, Clinical Disease Activity Score; CDAI-100, ≥100-point decrease in CDAI score; IFX, infliximab; IV, intravenous; PMS, partial Mayo score; QnW, every *n* weeks; SC, subcutaneous; SES-CD, Simplified Endoscopic Activity Score for Crohn’s disease; UC, ulcerative colitis.

## Supplementary Fig. 3 Mean (95% CI) CDAI score and PMS as efficacy parameters in patients with (a) CD and (b) UC


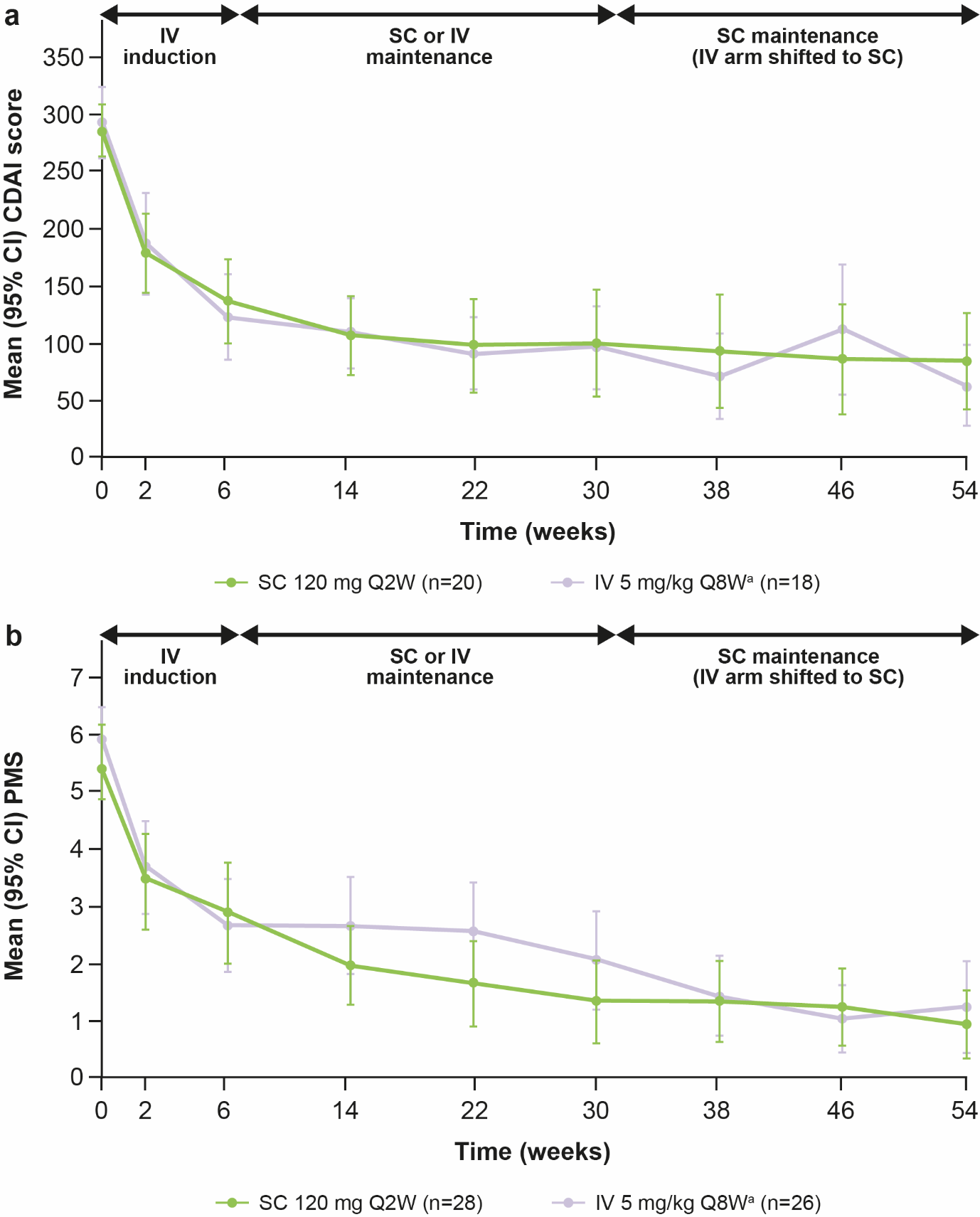


^a^Patients in the IV 5 mg/kg Q8W subset shifted to IFX SC 120 mg Q2W at Week 30.

Efficacy was analyzed in patients who received at least one full dose of study drug at Week 6 or thereafter and had at least one post-Week 6 efficacy assessment result.

CD, Crohn’s disease; CDAI, Clinical Disease Activity Index; CI, confidence interval; IFX, infliximab;
IV, intravenous; PMS, partial Mayo score; QnW, every n weeks; SC, subcutaneous; UC, ulcerative colitis.

## Supplementary Fig. 4 Mean (95% CI) levels of (a) FC and (b) CRP in patients with CD and UC (pooled)

**
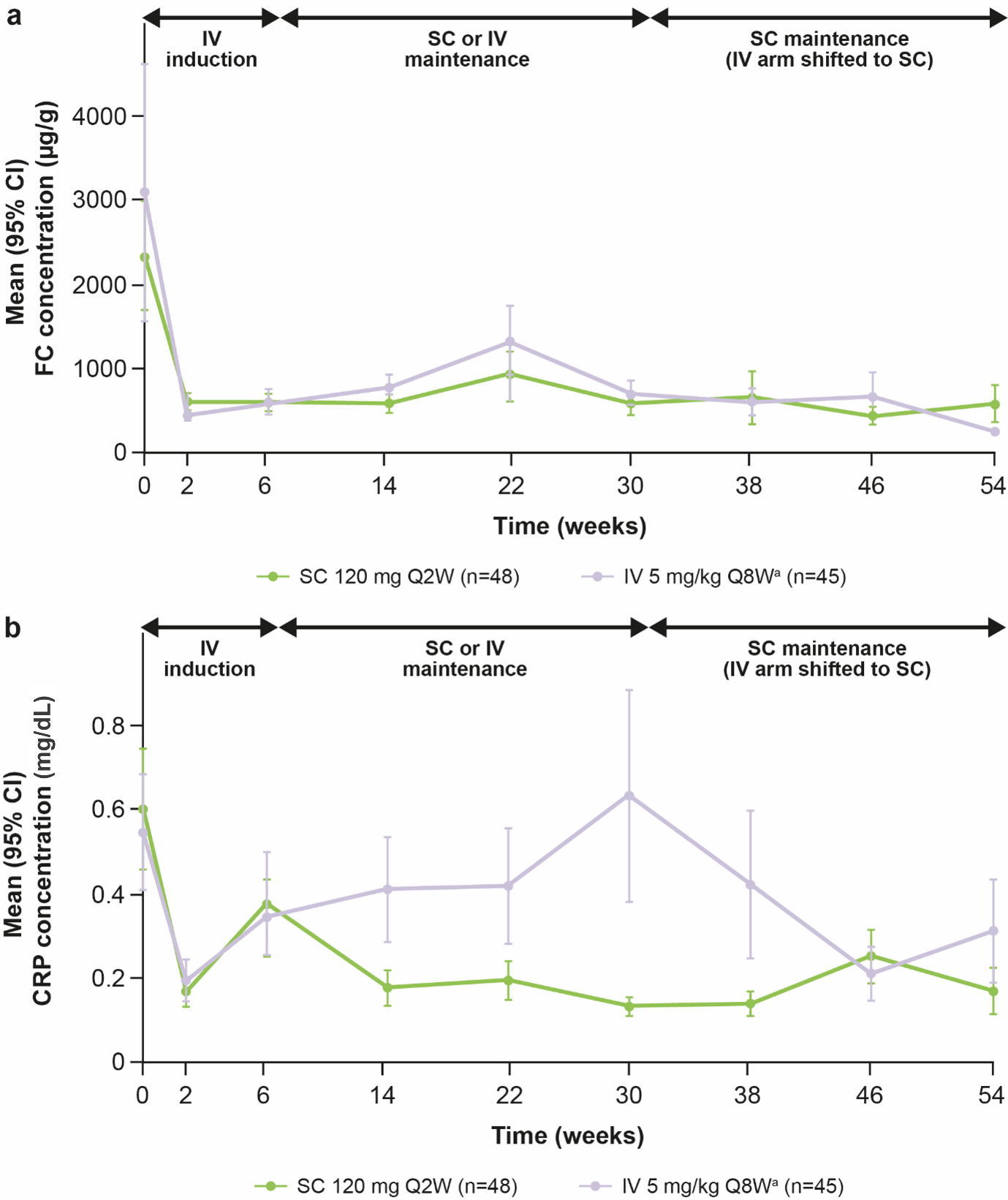
**

^a^Patients in the IV 5 mg/kg Q8W subset shifted to IFX SC 120 mg Q2W at Week 30.

CD, Crohn’s disease; CI, confidence interval; CRP, C-reactive protein; FC, fecal calprotectin; IFX, infliximab; IV, intravenous; QnW, every *n* weeks; SC, subcutaneous; UC, ulcerative colitis.

## Reference

1. Schreiber S, Ben-Horin S, Leszczyszyn J, Dudkowiak R, Lahat A, Gawdis-Wojnarska B, et al. Randomized controlled trial: subcutaneous vs intravenous infliximab CT-P13 maintenance in inflammatory bowel disease. Gastroenterology. 2021;160(7):2340–53. <https://doi.org/10.1053/j.gastro.2021.02.068>.
